# NF-kappa B interacting long noncoding RNA enhances the Warburg effect and angiogenesis and is associated with decreased survival of patients with gliomas

**DOI:** 10.1038/s41419-020-2520-2

**Published:** 2020-05-07

**Authors:** Zheng Chen, Shiting Li, Lin Shen, Xiangyu Wei, Hanshuo Zhu, Xueyi Wang, Min Yang, Xuesheng Zheng

**Affiliations:** 1 Department of Neurosurgery, XinHua Hospital, Shanghai JiaoTong University School of Medicine, 1665 KongJiang Rd, Shanghai, 200092 China; 2 The Cranial Nerve Disease Center of Shanghai JiaoTong University, Shanghai, 200092 China

**Keywords:** Cancer metabolism, CNS cancer

## Abstract

In various malignant tumors, NF-kappa B interacting long noncoding RNA (NKILA) displays antitumor activity by inhibiting the NF-kappa B pathway. However, the role of NKILA in gliomas remains unclear. Surprisingly, this study showed that NKILA is significantly upregulated in gliomas, and the increased levels of NKILA were correlated with a decrease in patient survival time. NKILA increased the expression level of hypoxia-inducible factor-1α, and the activity of the hypoxia pathway in gliomas. Furthermore, we demonstrated that NKILA enhances the Warburg effect and angiogenesis in gliomas both in vitro and in vivo. Therefore, NKILA is a potential therapeutic target in gliomas. In addition, we showed that a 20(S)-Rg3 monomer suppresses NKILA accumulation and reverses its stimulation of the Warburg effect and angiogenesis in gliomas, both in vitro and in vivo. Therefore, this study not only identified NKILA as a potential therapeutic target in gliomas, but also demonstrated a practical approach to treatment.

## Introduction

Long noncoding RNAs (lncRNAs) are noncoding chains of more than 200 ribonucleotides that play vital roles in various processes occurring in both normal and cancerous cells^1–3^. Recent research has shown that lncRNAs are abundantly expressed in human cells and frequently misregulated in malignant tumors. Consequently, some lncRNAs may be used as diagnostic and/or prognostic markers^4–6^. However, lncRNAs often achieve their cellular effects via novel mechanisms, and developing drugs that target these mechanisms may be challenging^7^.

NF-kappa B interacting lncRNA (NKILA), which was identified in 2015, is upregulated by NF-kappa B and binds strongly to NF-kappa B/IkB. NKILA masks IkB phosphorylation motifs, thereby stabilizing the NF-kappa B/IkB complex by inhibiting IkB phosphorylation and NF-kappa B activation^8^. Therefore, NKILA provides negative feedback to regulate NF-kappa B signaling. In many types of malignant tumors, such as breast cancer, rectal cancer, tongue squamous cell carcinoma, retinoblastoma, laryngeal cancer, malignant melanoma, and non-small cell lung cancer, NKILA suppresses NF-kappa B-mediated cancer metastasis. Therefore, NKILA acts as a tumor suppressor, and low levels of NKILA in patients predict poorer clinical outcomes^9–12^.

Gliomas are the most common malignant tumors of the human brain and are characterized by excessive proliferation, local invasion, high rates of recurrence, and poor prognoses^13,14^; however, gliomas seldom metastasize^14,15^. Therefore, understanding how NKILA functions in gliomas may be important. In this study, we found that NKILA is significantly upregulated in clinical glioma tissue samples and glioma cell lines. Furthermore, RNA profiling and clinical data from 669 patients with gliomas from The Cancer Genome Atlas (TCGA) database showed that high levels of NKILA correlated with high-grade glioblastomas and recurrent gliomas. In addition, in contrast to the types of cancer described above, high levels of NKILA were associated with decreased survival in patients with gliomas, suggesting that NKILA may be an important therapeutic target. To further investigate the role of NKILA in gliomas, we performed gene set enrichment analysis (GSEA) on the RNA sequencing (RNA-seq) profiles of the glioma cohort from TCGA database, and found that the level of NKILA positively correlated with hypoxia. Subsequent in vitro and in vivo experiments assessed the biological role of NKILA in gliomas, and integrative analysis revealed that NKILA may enhance the Warburg effect and angiogenesis in gliomas via the hypoxia pathway, thereby driving tumor progression. In addition, we show that a monomer from herbal medicine suppresses NKILA accumulation and reverses its stimulation of the Warburg effect and angiogenesis in gliomas. These observations may provide a basis for novel therapeutic strategies.

## Materials and methods

### Reagents, cell culture, and specimens

20(S)-ginsenoside-Rg3 (C42H72O13, 785.01 g/mol) was purchased from Tauto Biotech (Shanghai, China). Tumor necrosis factor alpha (TNF-α), 2-methoxyestradiol (2-ME) and Bevacizumab were purchased from MedChemExpress (Monmouth Junction, NJ, USA). GBM cell lines T98G, LN229, U87, U251, A172 were purchased and authenticated by Cell Bank of the Chinese Academy of Sciences (Shanghai, China). SVGp12, U373, U118, U138 were purchased from ATCC. The study protocols were approved by the Human Ethics Committee of Xinhua Hospital (Shanghai, China), and all patients provided informed consent. Tissue samples were obtained from 35 patients who underwent surgery in Xinhua Hospital between November 2017 and June 2019, and included 15 glioblastoma multiforme (GBM) samples, 10 low-grade glioma (LGG) samples, and 10 normal brain tissue samples (4 from craniocerebral trauma patients and 6 neighboring noncancerous tissue samples from glioma patients). All samples were verified by two independent pathologists.

The culture medium of U87 was composed of MEM (Gibco, NY, USA), 1%NEAA (Gibco, NY, USA) and 10% FBS (Gibco, NY, USA), 100 U/mL penicillin and 100 μg/mL streptomycin (Gibco, NY, USA). The others were cultured in DMEM high-glucose medium (Gibco, NY, USA) supplemented with 10% FBS (Gibco, Massachusetts, USA) at 37 °C in a humidified atmosphere of 5% CO_2_, 95% air and 100% humidity.

### Bioinformatics analysis

Clinical information and RNA-seq data from 669 patients with gliomas were obtained from TCGA (https://tcga-data.nci.nih.gov/tcga) and analyzed using R software (ver. R 3.5.0; R Development Core Team, Vienna, Austria). The relevance of NKILA level with the gender, age, pathological stage and recurrence was demonstrated using R package “ggstatsplot”. Then Kaplan–Meier survival curves of the NKILA-high and NKILA-low subsets were generated using R packages “survival”, “survminer” and “survivalROC”. Gene set enrichment analysis (GSEA) was performed to identify enriched pathways in NKILA-high or NKILA-low subsets using “clusterProfiler” package; if the absolute value of Normalized Enrichment Score (NES) of GSEA is more than 1, Nominal P-value less than 0.01 and False discovery rate (FDR) *q*-value less than 0.25, that gene set is considered as enriched.

### RNA interference and vectors

Three HIF-1a-siRNAs and their negative control (NC) siRNAs were purchased from RiboBio, China. The lentivirus vector containing three short hairpin RNAs (shRNA) knock-down NKILA (including sh-NKILA1, sh-NKILA2, sh-NKILA3), lentivirus knock-down Negative Control (sh-NC), lentivirus overexpress NKILA, lentivirus overexpress Negative Control were purchased from Cyagen (Guangzhou, China). Stably shRNA-NKILA-transfected cells were selected by the treatment of puromycin (1 μg/ml, Solarbio, China). The sequences of si-RNAs and sh-RNAs are listed in Supplementary Table S1. After RNA interference, lentivirus strains showing the greatest knockout efficiency were selected for follow-up experiments.

### RNA extraction and qRT-PCR

Total RNA was isolated from tumor tissues or cell lines using TRIzol (TaKaRa, Dalian, China). The PrimeScript™ one step RT-PCR kit (TaKaRa, Dalian, China) was used to reverse transcribe RNA into cDNAs according to the manufacturer’s protocol. And the mRNA level was measured using the SYBR^®^ Premix DimmerEraser™ kit (TaKaRa, Dalian, China) and the ABI7500 system (Applied Biosystems, Carlsbad, USA). β-actin was used as an internal control for normalization. The relative mRNA expression change was calculated by using 2^-ΔΔCt^ method. The real-time PCR were performed in triplicate. The primer sequences used were listed in Supplementary Table S2.

### Protein extraction and western blot analysis

Total protein was extracted with RIPA lysis buffer (Beyotime, China) supplemented with 0.01% protease and phosphatase inhibitor (Roche Applied Science, Switzerland). The cell lysate was added and incubated on ice for 30 min. After sonication, cell lysis was centrifuged 14,000 rpm at 4 °C for 10 min; and proteins in the supernatant were harvested for the subsequent experiments. Equal amounts of protein were separated by 8–15% SDS-gel electrophoresis and transferred to PVDF membranes (Millipore, USA). The primary antibodies used were listed in Supplementary Table S3. All experiments were performed in triplicate.

### Human umbilical vein endothelial cell (HUVEC) tube formation assay

The lentivirus-transfected cell lines (U87/A172-O.E.-NC, U87/A172-O.E.-NKILA, LN229/T98G-K.D.-NC, and LN229/T98G-K.D.-NKILA) were co-cultured with HUVECs for 24 h in 24-well co-culture plates (Corning Inc., Corning, NY, USA). The HUVEC tube formation assays were performed 24 h later, in accordance with the manufacturer’s instructions. Briefly, 80 μL of Matrigel (BD Biosciences, San Jose, CA, USA) was added to each well of a 96-well plate and incubated for 1 h at 37 °C with 5% CO_2_. During this incubation period, the co-cultured HUVECs were harvested and 1 × 10^4^ HUVECs in 100 μL of conditioned medium were added to each well and incubated at 37 °C in 5% CO_2_ for 3 h. Bright-field images were recorded using a microscope (Leica, Wetzlar, Germany). The extent of tube formation was compared to that of cell strings.

### Chicken chorioallantoic membrane (CAM) assays

CAM assays were performed on day 10 of the development of fertilized chicken eggs and used to evaluate direct effects on angiogenesis. A circular window with a diameter of 1 cm (located above the egg air chamber) was opened in the shell of each egg with a 10-day-old chicken embryo (Shanghai Veterinary Research Institut, Shanghai, China). To expose the CAM, the surface of the dermic sheet on the floor of the air chamber was removed. And filter paper with a diameter of 0.5 cm was placed on top of the CAM, and then media of lentivirus-transfected cell lines (50 μl, 1 × 10^6^) was added onto the center of the paper respectively. Then closed the window with sterile adhesive tape, and the eggs were incubated at 37 °C under 75% humidity for 48 h. Forty-eight hours later, the CAMs were fixed by stationary solution (methanol: acetone = 1:1) for 30 min, the CAMs were cut and harvested, and photos of each CAM were taken using a digital camera (Nikon, Tokyo Metropolis, Japan). The effect of angiogenesis was evaluated by the number of second- and third-order vessels in comparison with that treated with the vector-control group.

### Extracellular acidification rate (ECAR) and oxygen consumption rate (OCR) assays

ECAR and cellular OCR were assessed using a Seahorse XF glycolysis stress test kit (Seahorse Bioscience, Billerica, MA, USA) and a Seahorse XF Cell Mito stress test kit, respectively. Measurements were recorded using a Seahorse XF 96 extracellular flux analyzer. Experiments were performed according to the manufacturer’s instructions. In a nutshell, cells transfected with indicated constructs. 1×10^5^ cells per well were then seeded into a Seahorse XF 96 cell culture microplate for 12 h, and keep the number of cells in each group as similar as possible. Then the baseline measurements were taken for each group, and then for ECAR, at the specified point in time, glucose, oligomycin (the oxidative phosphorylation inhibitor), and 2-DG (the glycolytic inhibitor) were sequentially injected into each well; and for OCR, oligomycin, FCCP (p-trifluoromethoxy carbonyl cyanide phenylhydrazone; the reversible inhibitor of oxidative phosphorylation), and the mitochondrial complex I inhibitor rotenone plus the mitochondrial complex III inhibitor antimycin A (Rote/AA) were sequentially injected. Data were analyzed by Seahorse XF-96 Wave software. ECAR and OCR is reported in pmols/minute and mpH/min, respectively. The results were normalized to cell number. All experiments were performed in triplicate.

### Glucose uptake, pyruvate, lactate, ATP, PKM, and LDHA assays

Glucose Uptake Colorimetric Assay Kit, Pyruvate Colorimetric Assay kit, Lactate Colorimetric Assay Kit, ATP Colorimetric Assay kit, Pyruvate Kinase Activity Colorimetric Assay Kit and Lactate Dehydrogenase Activity Assay Kit are all from Biovison (San Francisco, USA). These assays were performed according to the manufacturer’s instructions and the previous literature^16^, and all results were normalized to cell number.

For glucose uptake assay, cells transfected with indicated constructs, and incubated in complete culture medium at 37 °C for 48 h. Then cells were harvested and 2 × 10^4^ cells were seeded into 96-well plate, incubated for 12 h. Cells were washed with PBS and starved for glucose by incubating with Krebs-Ringer-Phosphate-HEPES (KRPH) buffer containing 2% BSA for 1 h. 10 μl of 2-DG (10 mM) was added and incubated for 30 min. Cells were lysed with 90 ml of extraction buffer and then frozen/thawed once and heated at 85 °C for 40 min. and added 10 ml neutralization buffer to neutralize cell lysate. After centrifugation at 1.2 × 10^4^ rpm for 5 min, the supernatant was used for subsequent determination of glucose uptake by the Glucose Uptake Colorimetric Assay Kit. The glucose uptake was assessed by measuring the optical density (OD) at 412 nm in a microplate reader (BioTek, Winooski, VT, USA).

For pyruvate level assay, cells were transfected or infected as in glucose uptake assay. Cells were collected and extracted with 4 volume of the Pyruvate Assay Buffer. The cells were centrifuged at 1.2 × 10^4^ rpm for 10 min at 4 °C to remove insoluble material. Then the supernatant was assayed by Pyruvate Colorimetric Assay kit. The reaction system was incubated at room temperature for 30 min in dark and then detected at 570 nm wavelength.

For lactate production Colorimetric Assay, cells (2 × 10^5^) were seeded into a 12-well plate and in complete culture medium at 37 °C for 12 h. Then changed fresh culture medium without FBS and incubated at 37 °C for 1 h. And separated the supernatant for measurement of lactate production. The reaction system was incubated at room temperature for 30 min in dark and then detected at 450 nm wavelength. For ATP level Colorimetric Assay, Cells (1.0 × 10^6^) were collected and extracted in 100 μl of the ATP Assay Buffer. The cells were centrifuged at 1.2 × 10^4^ rpm for 5 min and the supernatant was separated and used for ATP determination. The reaction system was incubated at room temperature for 30 min in dark and then detected at 570 nm wavelength.

For pyruvate kinase activity assay, 1.0 × 10^6^ Cells were harvested and extracted with 4 volume of the Pyruvate Kinase Assay Buffer. Then cells were centrifuged at 1.2 × 10^4^ rpm for 10 min to get clear extract. After centrifugation, the supernatant was assayed by Pyruvate Kinase Activity Assay Kit. The reaction system was incubated at room temperature for 20 min in dark and then detected at 570 nm wavelength.

For lactate dehydrogenase activity assay, 5 × 10^5^Cells were collected and homogenized in 100 μl of the Lactate Dehydrogenase Assay Buffer. The cells were centrifuged at 1.2 × 10^4^ rpm for 15 min and the supernatant was assessed by Lactate Dehydrogenase Activity Assay Kit. The reaction system was incubated at room temperature for 20 min in dark and then detected at 450 nm wavelength.

All experiments were performed in triplicate.

### Animal xenografts and micro positron emission tomography (PET)/computed tomography (CT) imaging

Seven mice per group were used in the survival time experiments. Five mice per group were used in other experiments. Each 4-week-old male nude mouse was subcutaneously injected with LN229 or U87 cells (100 μl, 1 × 10^6^ cells) that expressed O.E./K.D.-NC or O.E./K.D.-NKILA. For the 20(S)-Rg3 group, LN229 cells (100 μl, 1 × 10^6^) were subcutaneously injected on day 0; then, from day 5, each mouse received an intraperitoneal injection containing 20(S)-Rg3 30 mg/kg, every day for 15 consecutive days. Tumor volumes were measured every 5 days (0.5 × length × width^2^). After 25 days, the mice were sacrificed and tumors were excised, weighed, and immunohistochemically stained.

Micro PET/CT imaging of mice was performed using an animal PET scanner (Siemens Preclinical Solutions, Knoxville, Tennessee, USA). Ten minute static PET scans were acquired at the time point of 1 h after injection of 3.7 MBq (100 μCi) ^18^F-FDG or via tail vein, and then take a 10 min micro-CT scan to obtain anatomic information. For the ^18^F-FDG scans, before tracer injection, mice were fasted for about 6 hours. mice were anesthetized with 1.5% isoflurane in oxygen at 2 L/min and kept warm with a temperature-controlled heating system during the entire imaging procedure. The images were reconstructed by a three-dimensional ordered subsets expectation maximum (3D OSEM) algorithm without attenuation or scattering correction and were then processed by using Siemens Inveon Research Workplace 3.0 (IRW 3.0). The 3D regions of interest (ROIs) were manually drawn over the tumor to obtain the maximum standardized uptake value (SUVmax). Given a tissue density of 1 g/cm^3^, these values were then divided by the injected activity to obtain an image ROI-derived mean and maximum percent injected dose per gram of body weight (%ID/gmax).

### Statistical analysis

All statistical analyses were performed using SPSS software (ver. 20.0; SPSS, Inc., Chicago, IL, USA). Data are expressed as means ± standard deviation. And graphs were generated using the GraphPad Prism software (ver. 5.0.1; La Jolla, CA). For relative data analysis, the mean value of control group is defined as 1% or 100%. Differences between two groups were compared with the unpaired Student’s *t* test. Differences between multiple groups were compared with one‐way analysis of variance (ANOVA). The Kaplan–Meier method was used to assess survival, and log-rank tests were used to determine significance. All tests were two-tailed and a *p*-value < 0.05 was considered statistically significant.

## Results

### The expression of NKILA is significantly upregulated and positively correlated with the activity of the hypoxia signaling pathway in gliomas

**Fig. 1:**
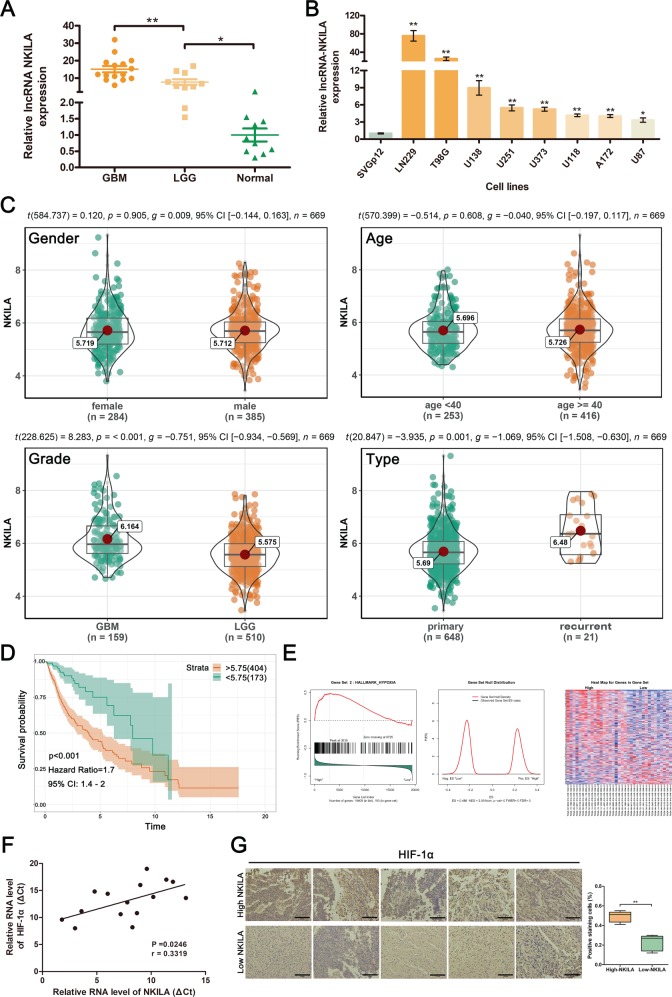
The expression of NF-kappa B interacting long noncoding RNA (NKILA) is significantly upregulated in gliomas and positively correlated with the activity of the hypoxia signaling pathway. **a** The relative expression of NKILA in clinical glioblastoma multiforme (GBM) tissues (*n* = 15), low-grade glioma tissues (*n* = 10), and normal brain tissues (*n* = 10) was assessed using quantitative real-time polymerase chain reaction (qRT-PCR) *n* = 4. The relative expression of NKILA was normalized using β-actin. **b** The relative expression of NKILA in glioma cell lines (LN229, T98G, U138, U251, U373, U118, A172, and U87) and the normal human astrocyte cell line SVGp12 was assessed using qRT-PCR (*n* = 4). **c** Clinical data from The Cancer Genome Atlas (TCGA) database representing 669 patients were used to investigate correlations among sex, age, tumor grade, tumor type, and the expression of NKILA in tumor tissue. **d** Kaplan–Meier overall survival curve for 669 glioma patients from TCGA database correlated with NKILA expression. **e** Gene set enrichment analysis (GSEA) plots show a significant correlation between NKILA expression levels in gliomas and gene signatures for hypoxia. **f** Spearman’s correlation between NKILA expression and the hypoxia-inducible factor (HIF)-1α mRNA level was assessed in 15 clinical GBM tissue samples (*r* = 0.5868, *p* = 0.0215). **g** Representative immunohistochemically stained micrographs showing HIF-1α expression in clinical GBM tissues with high and low NKILA mRNA levels. n = 3. Scale bar = 100 μm. Data are expressed as means ± standard deviation (SD) of triplicate experiments, *p < 0.05, **p < 0.01.

To investigate the role of NKILA in gliomas, we used qRT-PCR to measure the expression levels of NKILA in 15 clinical GBM tissue samples, 10 clinical LGG tissue samples, and 10 normal brain tissue samples. The expression levels of NKILA were significantly higher in the GBM and LGG samples than in the normal brain tissue samples (Fig. 1a). Moreover, the levels of NKILA in GBM tissues were higher than those in LGG tissues. In addition, we measured NKILA expression levels in eight human glioma cell lines, and in the normal astrocyte human cell line SVGp12. NKILA transcript levels were generally higher in the glioma cell lines than in SVGp12 (Fig. 1b). These results suggest that NKILA is highly expressed in gliomas.

We also analyzed the RNA profiles and clinical data of 669 patients with gliomas from TCGA database; the analyses indicated that NKILA expression was significantly correlated with tumor grade and type. High levels of NKILA expression were correlated with high-grade glioblastomas and recurrent gliomas. However, NKILA expression levels were unaffected by patient sex or age (Fig. 1c). In addition, Kaplan–Meier analyses indicated decreased survival times in patients with high levels of NKILA expression (Fig. 1d). Furthermore, GSEA of glioma RNA-seq profiles showed that NKILA expression was positively correlated with the activity of the hypoxia pathway (Fig. 1e). The qRT-PCR experiments showed that the expression of hypoxia-inducible factor (HIF)-1α was positively correlated with NKILA levels in 15 GBM tissues (*p* = 0.0246, *r* = 0.3319; Fig. 1f). We separated tissue from 15 GBM patients into high- (*n* = 8, NKILA expression ratio ≥ median ratio) and low-level NKILA expression groups (*n* = 7, NKILA expression ratio ≤ median ratio). Next, we used immunohistochemical staining to assess the HIF-1α expression levels in five samples randomly selected from each of these two groups. We found that the expression levels of HIF-1α in the high-level NKILA group were higher than those in the low-level NKILA group (Fig. 1g). In contrast to studies on other types of cancer, these data suggest that NKILA may stimulate tumor progression in gliomas.

### NKILA increases the expression of HIF-1α and activity of the hypoxia pathway in glioma cells under normoxic conditions

**Fig. 2:**
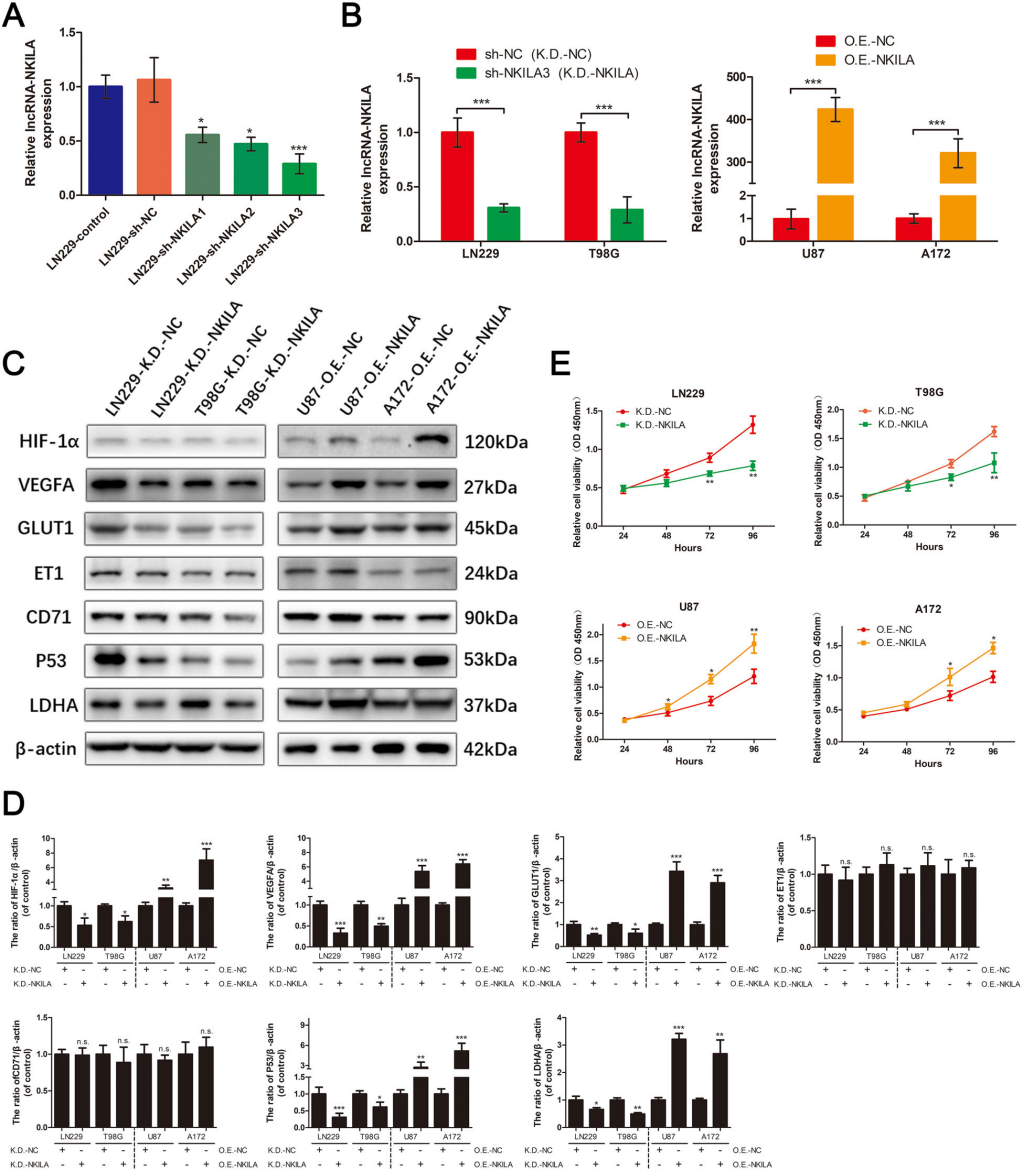
NKILA upregulates the expression of HIF-1α and the activity of the hypoxia pathway in glioma cells under normoxic conditions. **a** The expression of NKILA in cell line LN229 transfected with three different lentivirus knockdown NKILAs (sh-NKILA1/2/3) or lentivirus knockdown negative controls (sh-NC) was assessed using qRT-PCR (*n* = 4). **b** The expression of NKILA in cell lines LN229 and T98G transfected with sh-NKILA3 (K.D.-NKILA) or sh-NC (K.D.-NC) was assessed using qRT-PCR (*n* = 4). The expression of NKILA in cell lines U87 and A172 transfected with lentivirus overexpressing NKILA (O.E.-NKILA) or lentivirus overexpressing NC (O.E.-NC) was assessed using qRT-PCR (*n* = 4). **c**, **d** Western blotting analysis was used to measure protein levels of key molecules in the hypoxia pathway of LN229 and T98G cells transfected with K.D.-NKILA or K.D.-NC and U87/A172 cells transfected with O.E.-NKILA or O.E.-NC. *n* = 3. **e** Cell viability in K.D.-NKILA/NC-transfected LN229 and T98G or O.E.-NKILA/NC-transfected U87 and A172 cell lines was determined using Cell Counting Kit-8 assays (*n* = 6). Data are expressed as means ± SD of triplicate experiments, *p < 0.05, **p < 0.01, ***p < 0.001; n.s., not statistically significant.

To confirm that NKILA is involved in regulating the hypoxia pathway in glioma cells, we transfected glioma cells with lentivirus to downregulate or overexpress NKILA (Fig. 2a, b), and used western blotting analyses to assess changes in the expression of key proteins in the hypoxia pathway. We found that downregulation of NKILA in LN229 and T98G cells under normoxic conditions significantly inhibited the expression of the HIF-1α protein (Fig. 2c, d), whereas overexpression of NKILA in U87 and A172 cells under normoxic conditions significantly increased the expression of HIF-1α. We also found that the expression patterns of other key proteins in the hypoxia pathway showed the same changes in expression exhibited by HIF-1α. These proteins included vascular endothelial growth factor A (VEGFA), glucose transporter 1 (GLUT1), tumor suppressor protein 53 (p53), and LDHA. While, there was no obvious effect on ET1 and CD71, what are two potential target genes of HIF-1α^17,18^. In addition, previous studies have shown that activation of the hypoxia pathway in gliomas can promote cell proliferation^19,20^. Therefore, we used Cell Counting Kit-8 (Dojindo, Kumamoto, Japan) assays to evaluate whether NKILA could stimulate glioma cell proliferation in vitro. Decreasing the levels of NKILA significantly inhibited the proliferation of LN229 and T98G cells, whereas overexpression of NKILA stimulated the proliferation of U87 and A172 cells (Fig. 2e). These results suggest that under normoxic conditions, NKILA upregulates the expression of HIF-1α, and stimulates the hypoxia pathway and cell proliferation in vitro.

Additionally, Zhou et al. reported that hypoxia-induced NKILA expression negatively regulates NFκB to promote retinal pigment epithelium cells death^21^. This implies that there may be the possibility of the hypoxia-NKILA dual regulation theory. Here we carried out some experiments for a preliminary exploration. U87, A172, and LN229 cells were cultured in hypoxia for 24 h. Then, the mRNA and protein levels of HIF-1α in U87, A172, and LN229 cells under hypoxia or normoxia were determined by qRT-PCR and western blot assays. Meanwhile, the expression levels of NKILA in U87, A172, and LN229 cells under hypoxia or normoxia were quantified by qRT-PCR. As shown in Fig. S1a–c, the results showed that under hypoxia(24 h), the expression of HIF-1α increased dramatically, but NKILA did not change significantly in glioma cells. In addition, we used the CoCl_2_-hypoxia model to carry out the in vitro experiment once more (Fig. S1d–f). The results showed that there was a significant accumulation of HIF-1α protein in the cells, but the expression of NKILA was not upregulated in glioma. Therefore, at present, there is no evidence that acute hypoxia can lead to a significant upregulation of NKILA expression in gliomas.

### NKILA stimulates angiogenesis in gliomas in vitro and in vivo

**Fig. 3:**
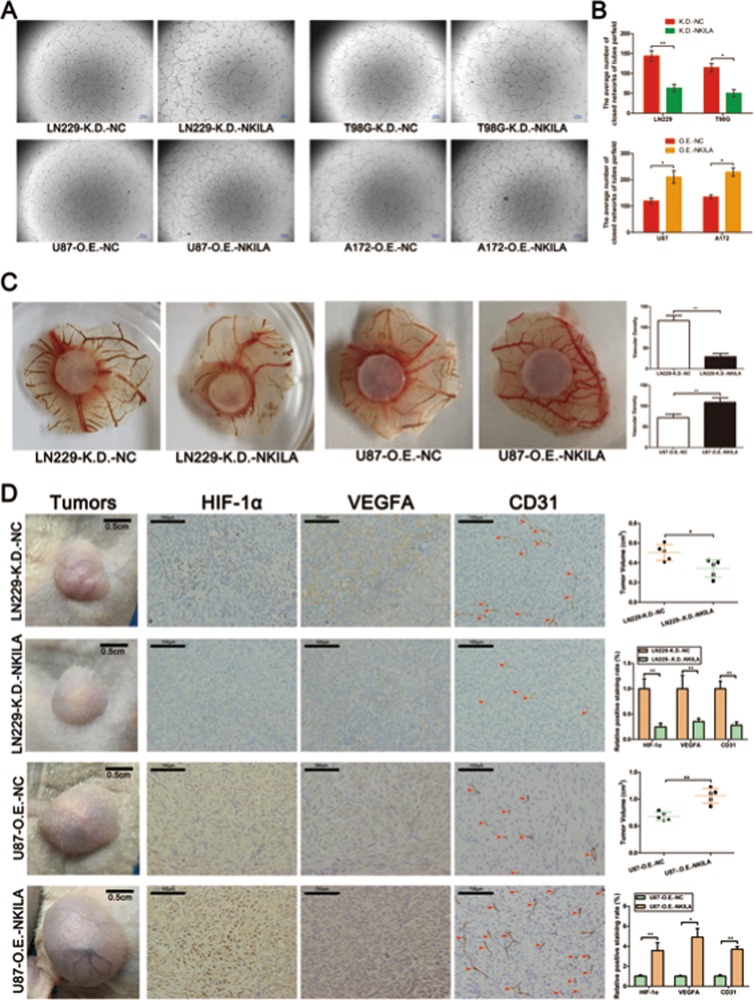
NKILA stimulates angiogenesis in gliomas both in vitro and in vivo. **a**, **b** The tube-forming capacity of glioma cells under different transfection conditions was determined using human umbilical vein endothelial cell (HUVEC) tube formation assays (*n* = 3). **c** Neovascularization in glioma cells under different transfection conditions was determined using chicken chorioallantoic membrane (CAM) assays n = 3. **d** Angiogenesis in vivo in glioma cells under different transfection conditions was determined using the subcutaneous xenograft model (*n* = 5) and HIF-1α-, vascular endothelial growth factor A (VEGFA)-, and CD31-immunohistochemical staining of subcutaneous xenograft tumor tissue *n* = 3. Data are expressed as means ± SD of triplicate experiments, *p < 0.05, **p < 0.01.

Many studies on human malignant neoplasms have reported that HIF-1α and VEGFA induce tumor angiogenesis^22,23^. Therefore, to investigate whether NKILA could stimulate angiogenesis in gliomas, we performed HUVEC tube formation assays. We found that downregulation of NKILA expression in glioma cells significantly decreased HUVEC tube formation in LN229 and T98G cell lines (Fig. 3a, b). In contrast, the upregulation of NKILA expression in tumor cells significantly increased angiogenesis in U87 and A172 cell lines. CAM assays showed similar results. The density of second- and third-order vessels in the CAMs of the LN229-K.D.-NKILA group was significantly lower than that of the LN229-K.D.-NC group. In contrast, the density of second- and third-order vessels in the CAMs of the U87-O.E.-NKILA group was significantly higher than that of the U87-O.E.-NC group (Fig. 3c).

To confirm the results of the in vitro studies, we investigated angiogenesis in glioma cells in vivo. LN229-K.D.-NC and LN229-K.D.-NKILA cells were subcutaneously injected into nude mice. We observed less tumor neovascularization in the LN229-K.D.-NKILA group than in the LN229-K.D.-NC group, whereas there was significantly more tumor neovascularization in the U87-O.E.-NKILA group than in the U87-O.E.-NC group (Fig. 3d). Moreover, immunohistochemical staining showed that tumor neovascularization index CD31 expression was significantly lower in LN229-K.D.-NKILA tumor tissue than in LN229-K.D.-NC tumor tissue, but significantly higher in the U87-O.E.-NKILA group than in the U87-O.E.-NC group. The expression levels of HIF-1α and VEGFA in tumor cells were similar to that of CD31.

In addition, to clarify the effect of NKILA on angiogenesis related to VEGFA secretion or to a direct effect on endothelial cells. we have carried out some experiments. Firstly, we overexpressed NKILA or NC in U87 and A172 cells with lentivirus (Lv-O.E.-NC or Lv-O.E.-NKILA), 48 h later, we determined the intracellular and extracellular (supernatant of glioma cells) NKILA levels in U87 and A172 cells with different transfection by qRT-PCR. As shown in Fig. S3a, the results showed that NKILA may not be released from inside to outside the cells, or it may be easily degraded outside the cells and unable to perform its biological function. Furthermore, we determined the cytokine VEGFA level in the medium of U87, A172, and LN229 cells transfected with Lv-O.E.-NC or Lv-O.E.-NKILA by Enzyme-linked immunosorbent assays (ELISA). As shown in Fig. S3b, ELISA assays showed that cytokine VEGFA level was markedly increased in U87, A172, and LN229 cells transfected with Lv-O.E.-NKILA supernatants. Additionally, we further verified the biological function of exocrine VEGFA in overexpressed NKILA glioma cells with Bevacizumab, which can specifically bind to VEGFA and block its biological function. As shown in Fig. S3c, the results of HUVEC (human umbilical vein endothelial cell) tube formation assays showed that when Bevacizumab blocked the biological function of VEGFA, glioma cells overexpressing NKILA could not improve the angiogenic ability of endothelial cells as well. Taken together, these data illustrated that the effect of NKILA on angiogenesis depend on the upregulated VEGFA secretion.

### NKILA modulates the Warburg effect in glioma cells in vitro

Several studies have reported that HIF-1α and p53 play important roles in the Warburg effect in tumor tissue^24,25^. In previous experiments, we found that NKILA can regulate the expression of HIF-1α and p53 in glioma cells. The expression of GLUT1, which plays a key role in regulating extracellular deoxyglucose uptake and LDH activity, showed similar alterations in expression (Fig. 2c). These results suggest that NKILA may modulate the Warburg effect in glioma cells.

**Fig. 4:**
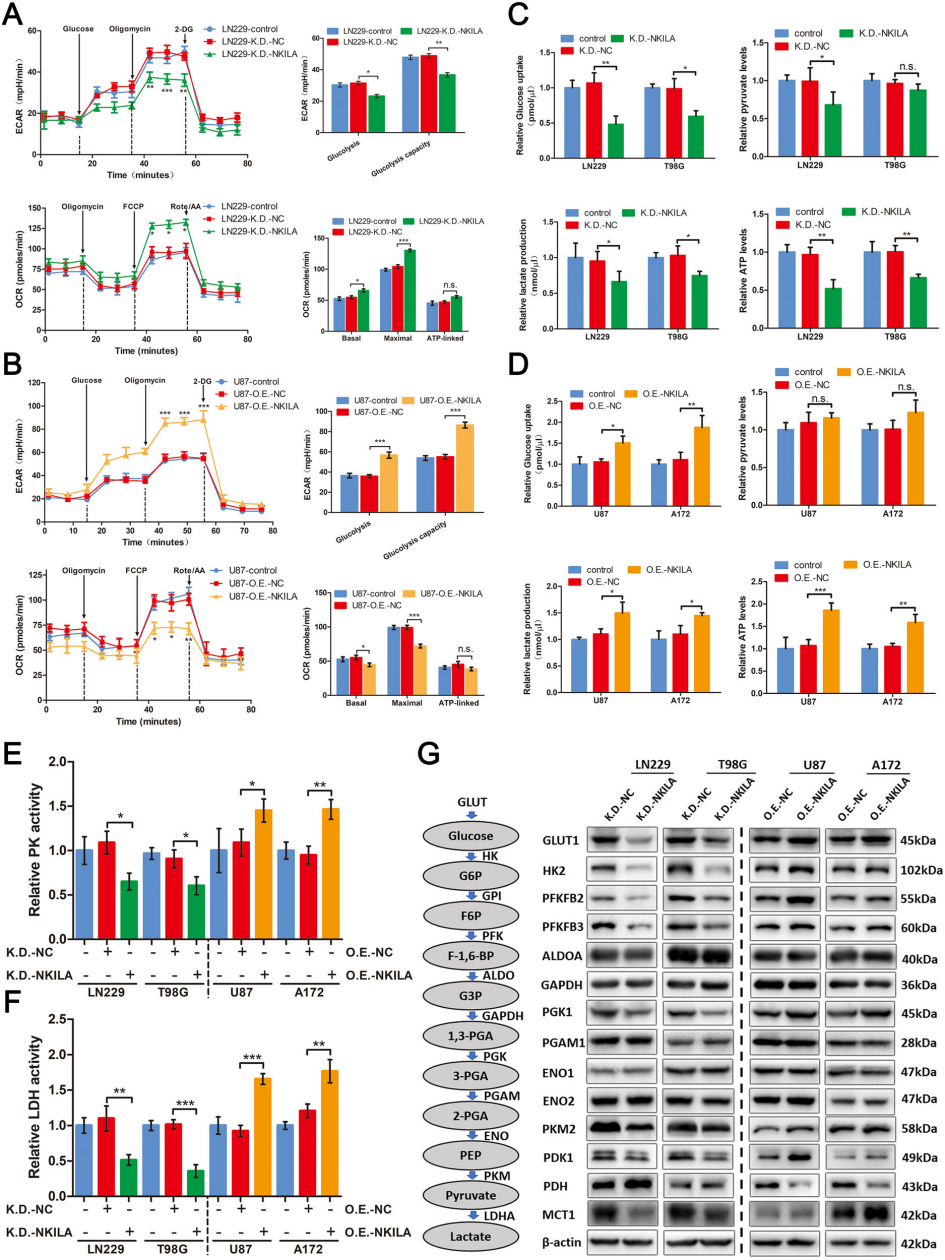
NKILA modulates the Warburg effect in glioma cells in vitro. **a**, **b** The extracellular acidification rate (ECAR) and oxygen consumption rate (OCR) were measured after LN229 cells were transfected with K.D.-NKILA or K.D.-NC. Similarly, ECAR and OCR were measured after U87 cells were transfected with O.E.-NKILA or O.E.-NC (*n* = 4). **c**, **d** The effects of different levels of NKILA expression on glucose uptake, pyruvate levels, lactate production, and adenosine triphosphate levels were determined using colorimetric assays after NKILA was downregulated in LN229 and T98G cells, or upregulated in U87 and A172 cells (*n* = 4). **e**, **f** The activities of the enzymes pyruvate kinase (PK) and lactate dehydrogenase (LDH) were determined in glioma cells under different transfection conditions using colorimetric assays (*n* = 4). **g** Western blotting analysis of glycolytic gene expression in LN229 and T98G cells transfected with K.D.-NKILA or K.D.-NC, as well as U87 and A172 cells transfected with O.E.-NKILA or O.E.-NC. *n* = 3. The aerobic glycolysis pathway is shown on the left. Data are expressed as means ± SD of triplicate experiments, *p < 0.05, **p < 0.01, ***p < 0.001; n.s., not statistically significant.

We performed a series of experiments on glioma cells to test this hypothesis. We tested the effect of NKILA on the overall regulation of glycolytic flux and mitochondrial respiration in glioma cells by measuring changes in ECAR and OCR, respectively. Compared to the LN229/T98G control group and the LN229/T98G-K.D.-NC group, the LN229/T98G-K.D.-NKILA group had a significantly lower ECAR and a higher OCR (Fig. 4a). In addition, the U87/A172-O.E.-NKILA groups showed increases in ECAR and decreases in OCR (Fig. 4b). We used colorimetric assays to evaluate key parameters of the Warburg effect in glioma cells, including glucose uptake, pyruvate levels, lactate production, and ATP generation. As expected, glucose uptake, lactate production, and ATP generation were significantly lower in LN229/T98G-K.D.-NKILA cells than in LN229/T98G control cells and LN229/T98G-K.D.-NC cells (Fig. 4c). In contrast, these three key parameters were significantly greater in U87/A172-O.E.-NKILA cells than in U87/A172 control cells and U87/A172-O.E.-NC cells (Fig. 4d). However, there were no consistent changes in pyruvate levels in glioma cells (Fig. 4c, d). Only LN229-K.D.-NKILA cells had pyruvate levels that were significantly lower than those of the control group, and there were no statistically significant differences in pyruvate levels among the T98G, U87, and A172 cell lines. This observation may reflect changes in pyruvate balance within glioma cells. Therefore, we assayed the activities of relevant enzymes within glioma cells, including PK, which catalyzes the conversion of phosphoenolpyruvate to pyruvate, and LDH, which catalyzes the conversion of pyruvate to lactic acid. When the expression of NKILA in LN229 and T98G cells was decreased, the activity of PK decreased markedly (Fig. 4e). When the expression of NKILA in U87 and A172 cells was increased, the activity of PK increased markedly. LDH activity was also positively correlated with the expression of NKILA in glioma cells (Fig. 4f). These data may explain why our colorimetric assays failed to detect changes in pyruvate levels in glioma cells: when the expression of NKILA was upregulated, the rate of pyruvate production increased, and the rate of conversion of pyruvate into lactic acid also increased.

Moreover, western blot analyses showed that, compared to LN229/T98G-K.D.-NC cells, LN229/T98G-K.D.-NKILA cells showed marked reductions in the expression of GLUT1, hexokinase 2 (HK2), 6-phosphofructo-2-kinase/fructose-2,6-bisphosphatase (PFKFB)2, PFKFB3, phosphoglycerate kinase 1, PK muscle isozyme 2 (PKM2), 3-phosphoinositide-dependent protein kinase-1, LDH, and monocarboxylate transporter 1 (MCT1), but not aldolase fructose-bisphosphate A, glyceraldehyde-3-phosphate dehydrogenase, phosphoglycerate mutase 1, enolase 1, or enolase 2. In addition, the expression of pyruvate dehydrogenase, which plays an important role in oxidative phosphorylation, increased (Fig. 4g). Similar results were observed in U87/A172-O.E.-NKILA cells compared to U87/A172-O.E.-NC cells. In addition, qRT-PCR showed changes in mRNA levels within T98G and A172 cells that were consistent with those observed in protein levels in the western blotting analyses (Fig. S2a, b). These data indicate that NKILA is involved in regulating glycolytic and oxidative phosphorylation-related gene expression in glioma cells. Therefore, NKILA modulates the Warburg effect in glioma cells in vitro.

**Fig. 5:**
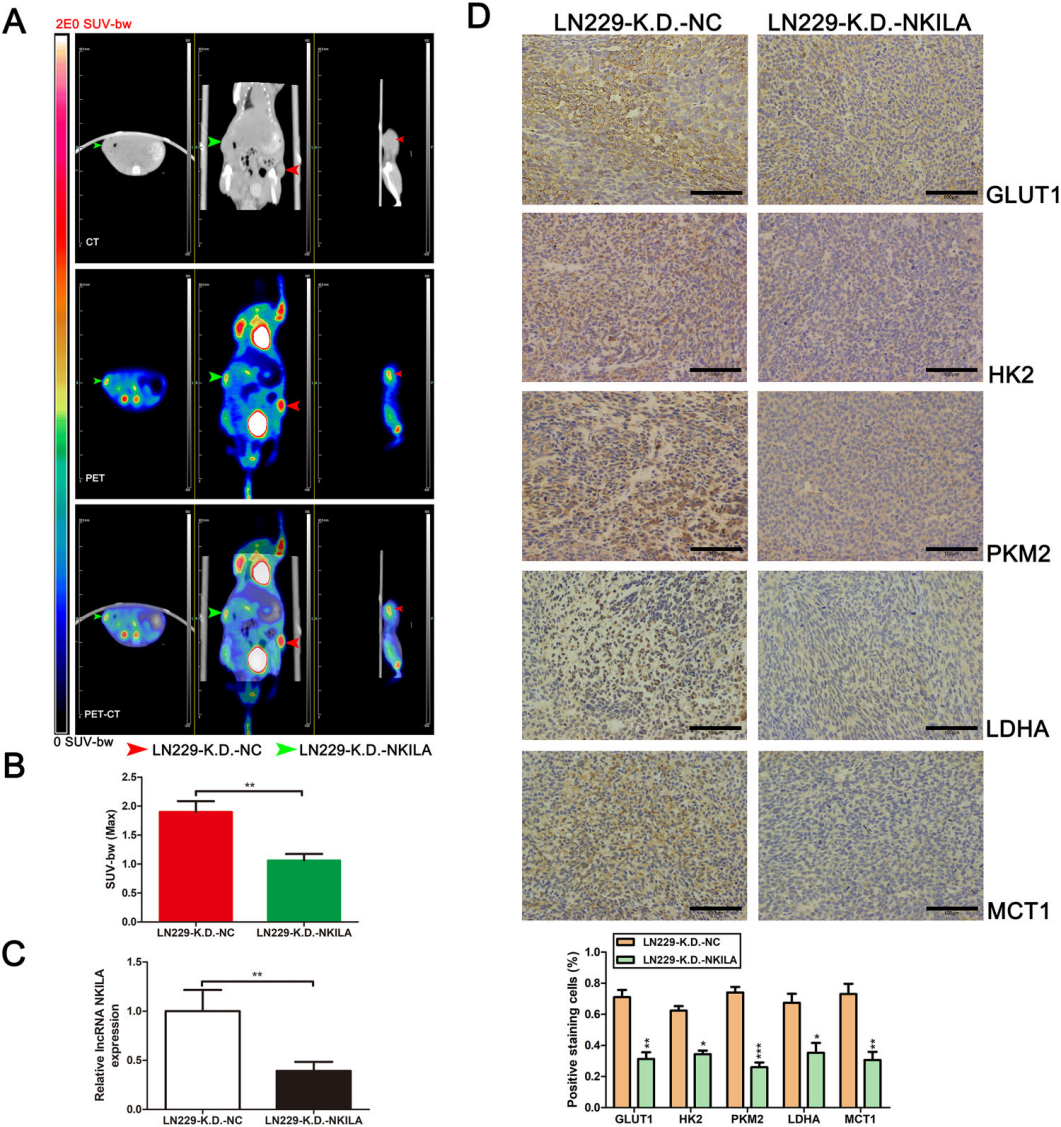
NKILA modulates the Warburg effect in glioma cells in vivo. **a**, **b** Representative 18F-fluorodeoxyglucose (^18^FDG) micro positron emission tomography (PET)/computed tomography (CT) images of nude mice injected with LN229-K.D.-NKILA (green arrows) or LN229-K.D.-NC (red arrows) cells. Glucose uptake (SUV-bw MAX) in the subcutaneous xenograft model (*n* = 5) is shown. **c** Relative expression of NKILA in tumor tissues from the LN229-K.D.-NKILA and LN229-K.D.-NC groups was assessed using qRT-PCR (*n* = 4). **d** The expression levels of glucose transporter 1 (GLUT1), hexokinase 2 (HK2), PK muscle isozyme 2 (PKM2), LDH, and monocarboxylate transporter 1 (MCT1) in tumors from the LN229-K.D.-NKILA and LN229-K.D.-NC groups were determined by immunohistochemical staining (n = 3). Data are expressed as means ± SD of triplicate experiments, **p < 0.01.

To investigate how NKILA modulates the Warburg effect in glioma cells in vivo, we subcutaneously injected LN229-K.D.-NKILA and LN229-K.D.-NC cells into nude mice. After 4 weeks, we used 18F-fluorodeoxyglucose (^18^FDG) microPET/CT to measure glucose uptake in tumor xenografts in the mice. The LN229-K.D.-NKILA group tumors showed significantly less glucose uptake than the LN229-K.D.-NC group tumors (Fig. 5a, b). qRT-PCR was used to analyze mouse tumor tissues and confirmed the effects of decreasing the levels of NKILA in the LN229-K.D.-NKILA group (Fig. 5c). In addition, immunohistochemical staining showed that GLUT1, HK2, PKM2, LDH, and MCT1 were significantly downregulated in LN229-K.D.-NKILA group tumors (Fig. 5d), and also confirmed that decreasing the levels of NKILA inhibited the Warburg effect in glioma cells in vivo, consistent with the in vitro results.

### NKILA stimulation of the Warburg effect and angiogenesis via HIF-1α activation in gliomas does not depend on the NF-kappa B pathway

**Fig. 6:**
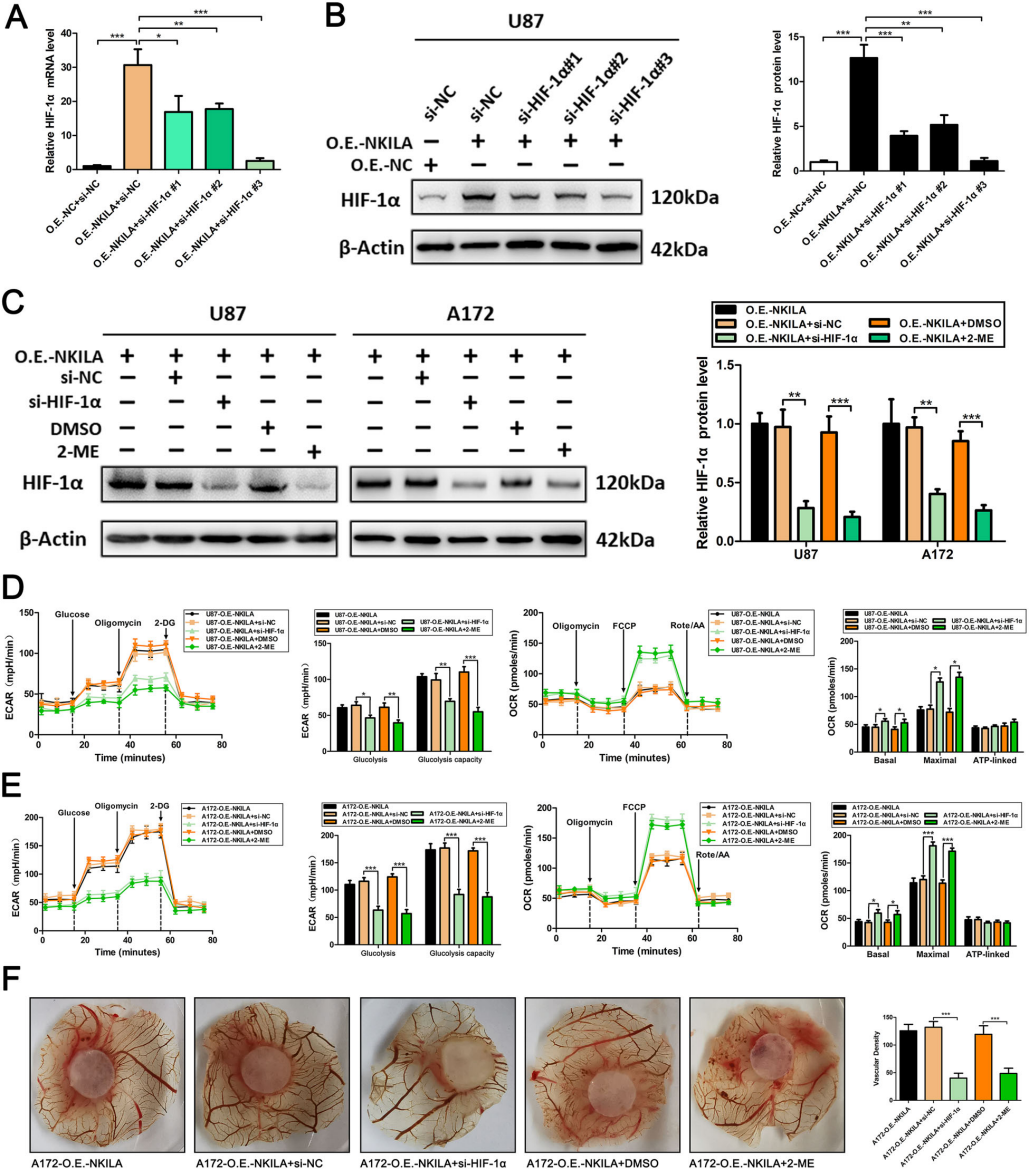
NKILA induces metabolic reprogramming and angiogenesis via HIF-1α activation in gliomas. **a**, **b** The mRNA and protein levels of HIF-1α in U87-O.E.-NKILA cells transfected with three different siRNAs against HIF-1α or si-NC were quantified by qRT-PCR (*n* = 4) and western blot assays (*n* = 3). *p < 0.05, **p < 0.01, ***p < 0.001 compared with control groups. **c** The inhibitory effect of si-HIF-1α #3 and 2-ME on the expression of HIF-1α protein in U87-O.E.-NKILA and A172-O.E.-NKILA cells were verified by western blot assays (*n* = 3). **p < 0.01, ***p < 0.001 compared with control groups. **d**, **e** The extracellular acidification rate and oxygen consumption rate were analyzed after the transfection of U87-O.E.-NKILA and A172-O.E.-NKILA cells with si-NC or HIF-1α or treated with DMSO or 2-ME by Seahorse XF (*n* = 4). **p < 0.01, ***p < 0.001 compared with si-NC or DMSO groups. **f** Representative images of the CAM blood vessels stimulated by glioma cells under different treatment (A172-O.E.-NKILA, A172-O.E.-NKILA + si-NC, A172-O.E.-NKILA + si-HIF-1α, A172-O.E.-NKILA + DMSO, and A172-O.E.-NKILA + 2-ME.). The assay was repeated at least three times with similar results. ***p < 0.001 compared with si-NC or DMSO groups. 2-ME, 2-methoxyestradiol; CAM, chicken chorioallantoic membrane.

To elucidate the role of HIF-1α in the effect of NKILA on GBM cell metabolic reprogramming and angiogenesis, we transfected three HIF-1α-specific siRNAs into U87 cells. As shown in Fig. 6a, b, interference of HIF-1α mRNA and protein levels was observed in siHIF-1α#3 cells, and we chose this siRNA for HIF-1α knockdown. And HIF-1α inhibitor 2-ME was parallel used for the subsequent experiments (Fig. 6c). The extracellular acidification rate and oxygen consumption rate were analyzed, and CAM assays were performed, as shown in Fig. 6d–f. The results showed that inhibition of HIF-1α expression in glioma cells with overexpression of NKILA can effectively reverse the stimulation of the Warburg effect and angiogenesis in gliomas. Therefore, these data furtherly indicated that NKILA modulates the Warburg effect of glioma cells via HIF-1α activation mediates the metabolic reprogramming.

**Fig. 7:**
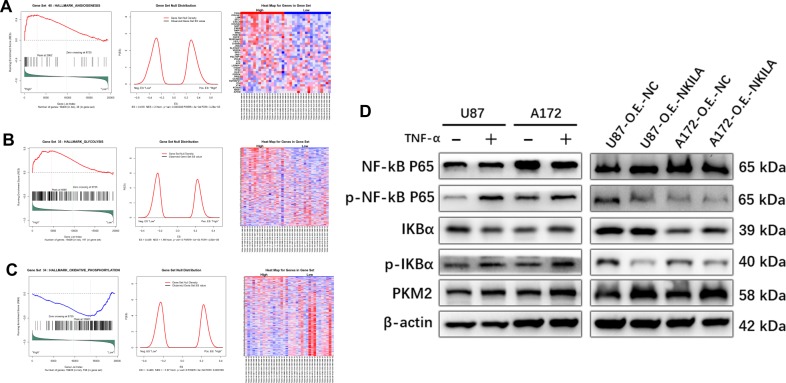
NKILA promotes the Warburg effect and angiogenesis in gliomas may not depend on the NF-kappa B pathway. **a** GSEA plots show a significant positive correlation between NKILA expression in gliomas and gene signatures for angiogenesis. **b**, **c** GSEA plots show that NKILA expression in gliomas is positively correlated with gene signatures for glycolysis and negatively correlated with gene signatures for oxidative phosphorylation. **d** The effects of NF-kappa B pathway activity and PKM2 protein levels on glioma cells. NKILA inhibits the NF-kappa B pathway. Western blotting analysis shows increased PKM2 protein levels in gliomas. The assay was repeated at least three times with similar results.

GSEA also showed that angiogenesis gene signatures were more prominent in NKILA-high expression than NKILA-low expression groups (Fig. 7a). Similar results were observed for glycolysis gene signatures, whereas oxidative phosphorylation gene signatures were less prominent in NKILA-high expression groups (Fig. 7b, c). Collectively, these data showed that NKILA overexpression contributes to angiogenesis and the Warburg effect in gliomas.

In various cancers, activation of the NF-kappa B pathway can promote the occurrence and development of tumors, including gliomas^26^. Azoitei et al.^27^ and Yang et al.^28^ reported that activation of the NF-kappa B pathway in tumor cells could promote tumor development by upregulating PKM2. Therefore, we performed western blotting analysis after U87 and A172 cells were treated with 20 ng/mL of TNF-α for 24 h (Fig. 7d). TNF-α activated the NF-kappa B pathway and significantly upregulated the expression of PKM2 in glioma cells.

NKILA can also inhibit IkB phosphorylation and NF-kappa B activation in many types of malignant tumors, but its effects in gliomas remain unclear. Western blotting showed that when NKILA was overexpressed in U87 and A172 cells, the activity of the NF-kappa B pathway in glioma cells decreased significantly. Interestingly, the expression of PKM2 increased significantly (Fig. 7d). These observations indicate that although NKILA provides negative feedback to regulate the NF-kappa B pathway in glioma cells, this pathway does not play a key role in promoting the Warburg effect or angiogenesis in gliomas.

### 20(S)-Rg3 reverses angiogenesis and the Warburg effect in gliomas by inhibiting NKILA expression in vitro and in vivo

**Fig. 8:**
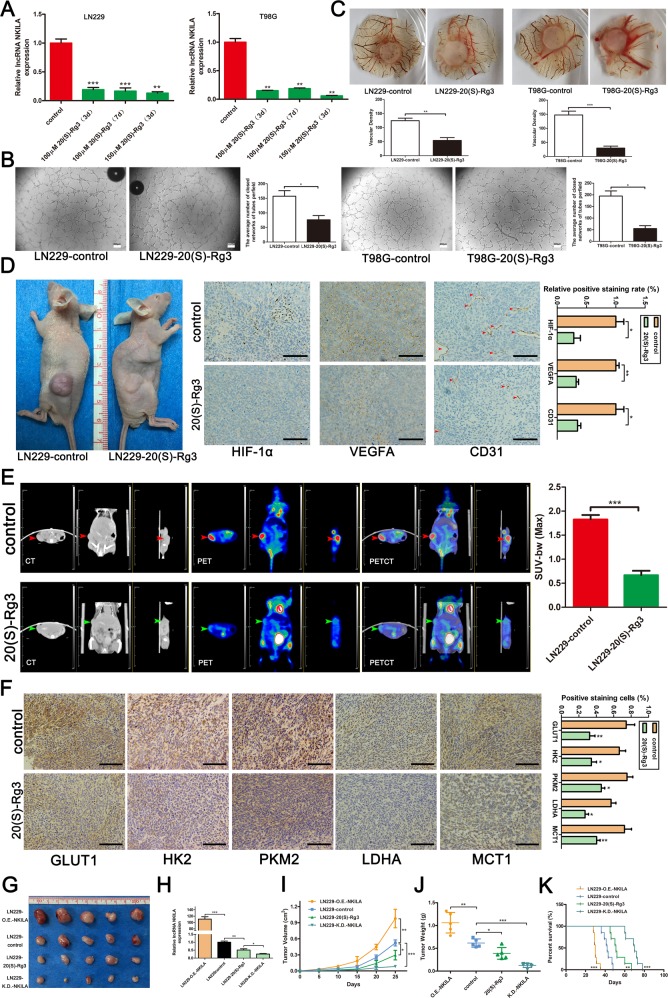
20(S)-Rg3 reverses angiogenesis and the Warburg effect in gliomas by inhibiting NKILA expression in vitro and in vivo. **a** LN229 and T98G cells were treated with 20(S)-Rg3 (100 μmol/L for 3 days, 100 μmol/L for 7 days, and 150 μmol/L for 3 days). Then, the expression level of NKILA was assessed using qRT-PCR. **b** The tube-forming capacity of LN229 and T98G cells after treatment with 20(S)-Rg3 (150 μmol/L for 3 days) was determined using HUVEC tube formation assays. **c** Neovascularization in LN229 and T98G cells treated with 20(S)-Rg3 (150 μmol/L for 3 days) was determined using chicken CAM assays. **d** Angiogenesis in vivo in LN229 cells treated with 20(S)-Rg3 was determined using the subcutaneous xenograft model and HIF-1α-, VEGFA-, and CD31-immunohistochemical staining of subcutaneous xenograft tumor tissue. **e** Representative ^18^FDG microPET/CT images of subcutaneous xenograft tumors (LN229 cells, five mice per group) in nude mice treated with 30 mg/kg 20(S)-Rg3 for 15 consecutive days (green arrows) or phosphate-buffered saline (PBS; red arrows). Glucose uptake (SUV-bw MAX) in the subcutaneous xenograft model (five mice per group) is shown. **f** The expression levels of glucose GLUT1, HK2, PKM2, LDH, and MCT1 in tumors after treatment with 20(S)-Rg3 (30 mg/kg for 15 consecutive days) or PBS were determined by immunohistochemical staining. **g**–**j** Subcutaneous tumor growth rates (five mice per group) in LN229 cells with different levels of NKILA expression (LN229-O.E.-NKILA, LN229-control, LN229-20(S)-Rg3, and LN229-K.D.-NKILA). The tumor growth curves are summarized in a line chart. Average tumor weights in the subcutaneous xenograft model are shown. **h** Relative expression of NKILA in tumors after different treatment in vivo was assessed using qRT-PCR (n = 4). **k** The survival of subcutaneous tumor-bearing mice from four different groups (LN229-O.E.-NKILA, LN229-control, LN229-20(S)-Rg3, and LN229-K.D.-NKILA; *n* = 7) is shown in a Kaplan–Meier plot. Data are expressed as means ± SD of triplicate experiments, *p < 0.05, **p < 0.01, *** *p* < 0.001.

In our previous study, we found that 20(S)-Rg3 could reverse temozolomide resistance in glioblastomas^29^. Interestingly, we also observed that 20(S)-Rg3 significantly suppressed the expression of NKILA in glioma cells. qRT-PCR showed that NKILA expression in glioma cell lines was suppressed by 20(S)-Rg3 by as much as 80% (Fig. 8a). We performed several assays to validate the effects of 20(S)-Rg3 on angiogenesis and the Warburg effect in gliomas. HUVEC assays showed that treating glioma cell lines LN229 and T98G with 150 μM 20(S)-Rg3 for 3 days resulted in a significant decrease in tube formation (Fig. 8b). The results of CAM assays were consistent with those of the HUVEC tube formation assays (Fig. 8c). To further investigate the inhibitory effect of 20(S)-Rg3 on NKILA expression in glioma cells in vivo, LN229 cells were subcutaneously injected into nude mice. Then, after 10 days, intraperitoneal injection of 30 mg/kg 20(S)-Rg3 or, an equal quantity of phosphate-buffered saline as a control, was administered every day for 15 consecutive days. We observed that the extent of neovascularization on the surface of subcutaneous tumors in the 20(S)-Rg3 group was significantly less than that observed in the control group after 15 days. Moreover, immunohistochemical staining showed a marked decrease in HIF-1α, VEGFA, and CD31 expression in 20(S)-Rg3 group tumor tissues (Fig. 8d). In addition, ^18^FDG microPET/CT scans for the two groups showed that glucose uptake in the 20(S)-Rg3 group tumors was significantly lower than that observed in the control group tumors (Fig. 8e). Furthermore, immunohistochemical staining showed that GLUT1, HK2, PKM2, LDH, and MCT1 were significantly downregulated in the 20(S)-Rg3 group, confirming that downregulation of NKILA by 20(S)-Rg3 reversed the Warburg effect in glioma cells in vivo (Fig. 8f). These observations are consistent with the experiments showing that lentivirus downregulated NKILA expression in LN229 cells.

We also compared the occurrence of tumorigenesis in vivo among LN229 cell lines with different levels of NKILA expression (Fig. 8g–k). These cell lines included the LN229-O.E.-NKILA, LN229-control, LN229-20(S)-Rg3, and LN229-K.D.-NKILA lines. QRT-PCR was used to analyze mouse tumor tissues and confirmed the effects of NKILA suppression by different treatment in vivo (Fig. 8h). Tumors in the LN229-O.E.-NKILA group developed substantially faster than those in the LN229-control group, whereas tumors in the LN229-K.D.-NKILA and LN229-20(S)-Rg3 groups developed more slowly than those in the LN229-control group (Fig. 8i). In addition, tumors in the LN229-O.E.-NKILA group were significantly heavier than those in the LN229-control group, whereas tumors in the LN229-K.D.-NKILA and LN229-20(S)-Rg3 groups weighed less than those in the LN229-control group (Fig. 8j). Moreover, decreasing the levels of NKILA significantly prolonged the median survival time of tumor-bearing nude mice, and the median survival time of LN229-20(S)-Rg3 tumor-bearing nude mice was significantly longer than that of LN229-control mice (Fig. 8k). These results indicate that 20(S)-Rg3 may reverse angiogenesis and the Warburg effect in gliomas by inhibiting NKILA expression in vitro and in vivo.

## Discussion

Gliomas are the most common malignant intracranial tumors and progress rapidly. Patients with gliomas, especially World Health Organization grade IV glioblastomas, have poor prognoses. Currently, treatment involves maximal surgical resection with post-operative adjuvant ionizing radiation and chemotherapy. However, the mean survival time of patients with glioblastomas is only 14.6 months^30,31^. Consequently, there is an urgent need for novel therapeutic strategies.

Whole-genome sequencing studies have shown that lncRNAs are frequently misregulated in malignant tumors, such that these lncRNAs are potential targets for treatment^7,32^. However, obtaining clinical solutions based on research findings is often difficult because the virus- and liposome-mediated transfection methods used to modulate lncRNA expression levels in the laboratory may not be practical in the clinic^7,33^.

Although NKILA acts as a tumor suppressor and inhibits metastasis in many other types of malignant cancers^34,35^, we found that NKILA stimulates the Warburg effect and angiogenesis, thus may promote the cell proliferation in gliomas, and that increased expression of NKILA was correlated with decreased patient survival time. Therefore, NKILA is a potential therapeutic target in gliomas. In addition, we found that the monomer 20(S)-Rg3 suppresses NKILA expression, and reverses its stimulation of the Warburg effect and angiogenesis. Therefore, this study not only identified NKILA as a potential therapeutic target in gliomas, but also demonstrated a practical approach to treatment.

NKILA was originally identified as an NF-kappa B-interacting lncRNA^8^. However, the functions of NKILA may not be restricted to the NF-kappa B pathway. For example, Lyu et al. reported that NKILA inhibits retinoblastoma by downregulating another lncRNA, X-inactive specific transcript^36^. NKILA may also interact with prostate transmembrane protein, androgen-induced 1 (PMEPA1), a gene that corresponds to part of the antisense sequence of NKILA. The expression of PMEPA1 is associated with several tumors, and this gene encodes a protein that regulates immune responses^37^. In this study, we found that NKILA stimulates the Warburg effect and angiogenesis in gliomas independent of the NF-kappa B pathway.

Our results are consistent with data obtained by GSEA of TCGA database showing that NKILA stimulates hypoxia, glycolysis, and angiogenesis, but suppresses oxidative phosphorylation. In particular, the expression of HIF-1α is upregulated in tumor cells overexpressing NKILA, and downregulated in tumor cells with low levels of NKILA expression. NKILA may initially interact with HIF-1α and subsequently influence other cellular processes. However, further studies are needed to test this hypothesis.

In normal brain cells, glucose metabolism proceeds predominantly by oxidative phosphorylation, and glycolysis is almost undetectable. The complete oxidation of one molecule of glucose can produce 38 ATP molecules, providing sufficient energy for brain function^38^. However, in gliomas, glucose metabolism is reprogrammed. Tumor cells exhibit drastically increased rates of glucose uptake and glycolysis, even when there is sufficient oxygen and the mitochondria are functioning normally. Glycolysis produces many intermediate metabolites, which are used in biosynthetic pathways to supply the tumor with amino acids and nucleotides for cell division. This reprogrammed metabolism is called the Warburg effect^25,39,40^ and provides malignant glioma cells with energy and fundamental building blocks^41^. In recent years, more and more glioma researchers have focused their attention on finding effective drugs targeting the Warburg effect of glioma cells^42,43^. Pre-clinical studies of 20(S)-Rg3 are needed to investigate its role in inhibiting the Warburg effect.

In addition, angiogenesis is an important therapeutic target in high-grade gliomas. Bevacizumab, a humanized monoclonal antibody against VEGFA, has been approved by the US Food and Drug Administration agency for use in the treatment of glioblastomas, especially for patients experiencing tumor recurrence following the standard Stupp regimen^44,45^. When the blood and oxygen supply decreases after bevacizumab treatment, the Warburg effect may be enhanced in the tumor as a compensatory mechanism^46^. Therefore, a combination of bevacizumab and 20(S)-Rg3 may have synergistic effects.

## Conclusions

This study showed for the first time that the lncRNA NKILA is upregulated in glioma tissues and cells, and increased expression of NKILA may be correlated with a poorer prognosis in patients with gliomas. NKILA stimulates activity of the hypoxia signaling pathway, the Warburg effect, and angiogenesis in gliomas, whereas 20(S)-Rg3 suppresses the expression of NKILA and reverses its stimulation of the Warburg effect and angiogenesis in gliomas. Our study of gliomas suggests that NKILA is a potential therapeutic target and 20(S)-Rg3 is a potential therapeutic agent.

## Supplementary information

Supplementary Figure Legends

Figure S1. NKILA may not be up-regulated in acute hypoxia in gliomas.

Figure S2. NKILA positively regulates the mRNA expression level of warburg effect related genes in glioma.

Figure S3. The effect of NKILA on angiogenesis depend on the up-regulated VEGFA secretion.

Supplementary Tables
