# Supplementary material for: NF-kappa B interacting long noncoding RNA enhances the Warburg effect and angiogenesis and is associated with decreased survival of patients with gliomas
Source: Cell Death Dis. 2020 May 7;11(5):323. doi: 10.1038/s41419-020-2520-2 (PMC7206073; doi:10.1038/s41419-020-2520-2)
Supplement: Supplementary file 1 — Supplementary Figure Legends [file 41419_2020_2520_MOESM1_ESM.docx]

**Supplementary Figure Legends**

**Figure S1**. **NKILA may not be up-regulated in acute hypoxia in gliomas. a-b.** The mRNA and protein levels of HIF-1α in U87, A172 and LN229 cells under hypoxia (for 24 hours) or normoxia were determined by qRT-PCR (n = 4) and western blot assays (n = 3). **p* < 0.05, ***p*<0.01, ****p*<0.001 compared with control groups. **c.** The expression levels of NKILA in U87, A172 and LN229 cells under hypoxia (for 24 hours) or normoxia were quantified by qRT-PCR (n = 4). **d-e.** The mRNA and protein levels of HIF-1α in U87, A172 and LN229 cells under treatment of CoCl_2_ (300 μM, for 6 hours) or DMSO as control were assessed by qRT-PCR (n = 4) and western blot assays (n = 3). ***p*<0.01, ****p*<0.001 compared with control groups. **f.** The expression levels of NKILA in U87, A172 and LN229 cells under treatment of CoCl_2_ (300 μM, for 6 hours) or DMSO were quantified by qRT-PCR (n = 4). n.s., not statistically significant.

**Figure S2. NKILA positively regulates the mRNA expression level of warburg effect related genes in glioma.** In parallel with the detection of changes in protein expression levels, we also detected changes in mRNA expression levels of warburg effect related genes by qRT-PCR(n=4) in T98G and A172 cells, and as shown in the Figure S2**a-b** the results are consistent with those of changes in protein expression levels. Compared with T98G-K.D.-NC cells, mRNA expression levels of GLUT1, HK2, PFKFB2, PFKFB3, PGK1, PKM2, PDK1, LDHA and MCT1 were significantly decreased in T98G-K.D.-NKILA group. Meanwhile, mRNA expression levels of these genes in A172-O.E.-NKILA group were significantly increased compared to A172-O.E.-NC cells. **p* < 0.05, ***p*< 0.01, *** *p*< 0.001, n.s., not statistically significant

**Figure S3. The effect of NKILA on angiogenesis depend on the up-regulated VEGFA secretion. a.** NKILA levels of intracellular and extracellular in U87 and A172 cells transfected with Lv-O.E.-NC or Lv-O.E.-NKILA were determined by qRT-PCR (n=4). ****p*<0.001 compared with control groups. **b.** The cytokine VEGFA level in the medium of U87, A172 and LN229 cells transfected with Lv-O.E.-NC or Lv-O.E.-NKILA were revealed by ELISA assay (n=4). **p* < 0.05, ***p*<0.01 compared with control groups. **c.** The tube-forming capacity of glioma cells under different transfection and treatment conditions were determined using HUVEC tube formation assays (n=3). ***p*<0.01 compared with control groups. n.s., not statistically significant. ELISA, Enzyme-linked immunosorbent assay. HUVEC, human umbilical vein endothelial cell.
