# Supplementary figures and images for: NF-kappa B interacting long noncoding RNA enhances the Warburg effect and angiogenesis and is associated with decreased survival of patients with gliomas
Source: Cell Death Dis. 2020 May 7;11(5):323. doi: 10.1038/s41419-020-2520-2 (PMC7206073; doi:10.1038/s41419-020-2520-2)

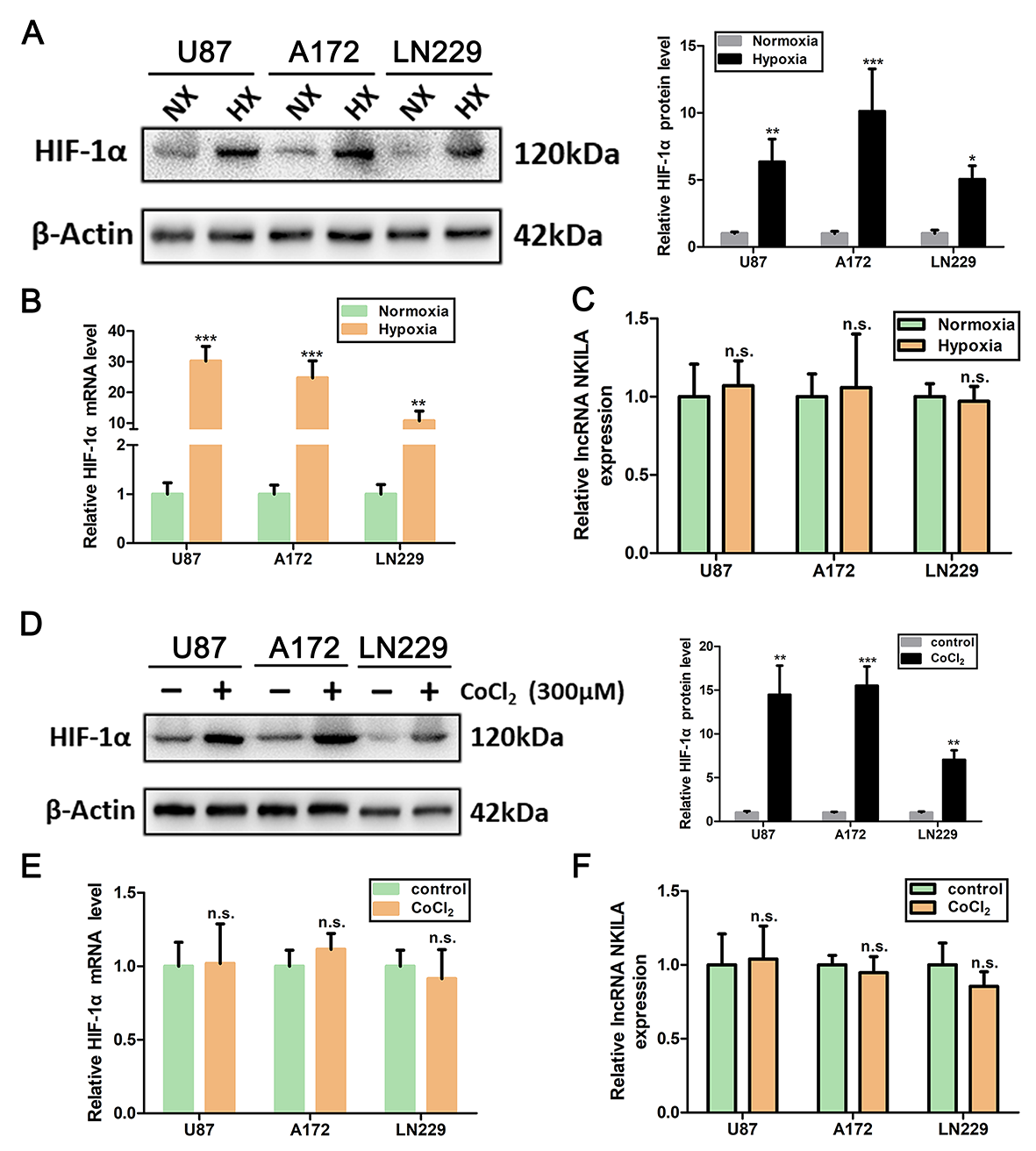

Supplement: Supplementary file 2 — Figure S1. NKILA may not be up-regulated in acute hypoxia in gliomas. [file 41419_2020_2520_MOESM2_ESM.tif]

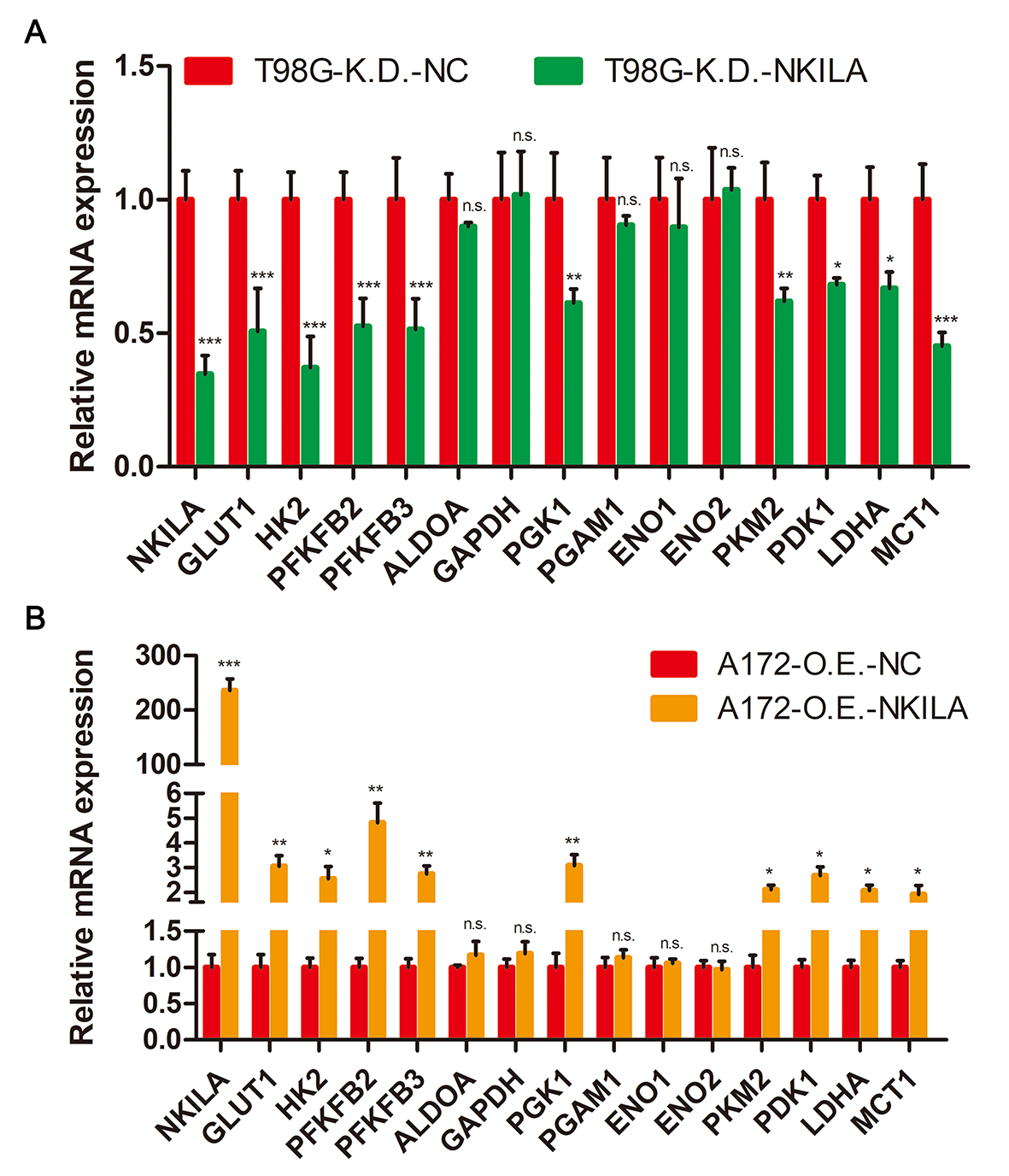

Supplement: Supplementary file 3 — Figure S2. NKILA positively regulates the mRNA expression level of warburg effect related genes in glioma. [file 41419_2020_2520_MOESM3_ESM.tif]

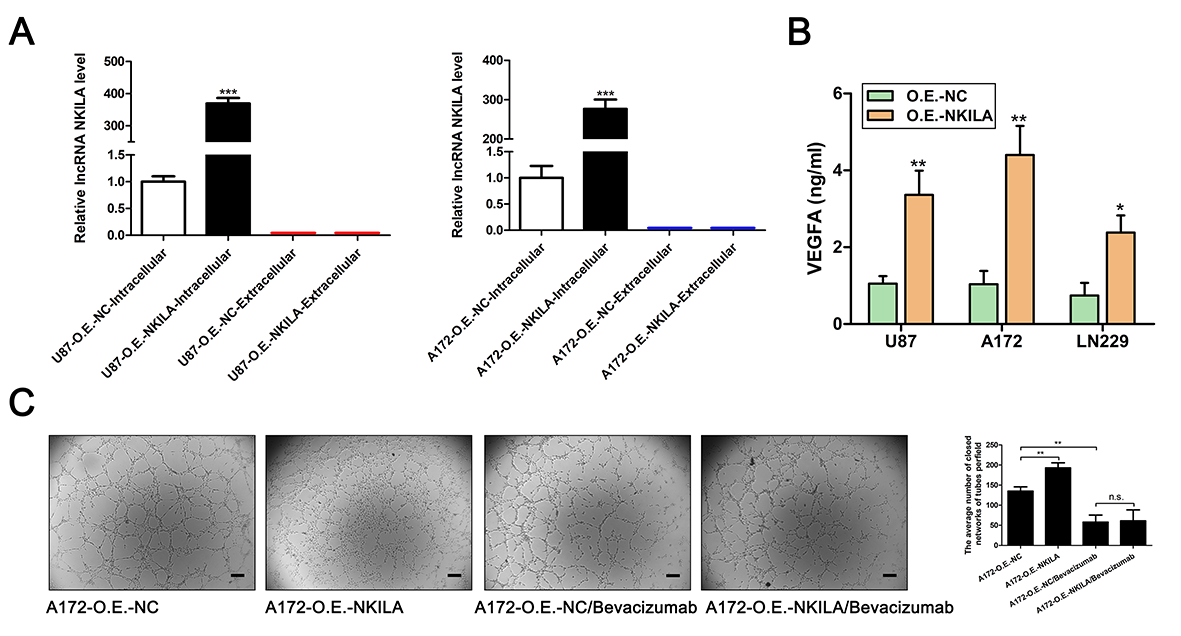

Supplement: Supplementary file 4 — Figure S3. The effect of NKILA on angiogenesis depend on the up-regulated VEGFA secretion. [file 41419_2020_2520_MOESM4_ESM.tif]
