# Supplementary material for: NF-kappa B interacting long noncoding RNA enhances the Warburg effect and angiogenesis and is associated with decreased survival of patients with gliomas
Source: Cell Death Dis. 2020 May 7;11(5):323. doi: 10.1038/s41419-020-2520-2 (PMC7206073; doi:10.1038/s41419-020-2520-2)
Supplement: Supplementary file 5 — Supplementary Tables [file 41419_2020_2520_MOESM5_ESM.docx]

**Supplementary Tables.**

| **Table S1. shRNA and si-RNA sequences** | | | | | |
| --- | --- | --- | --- | --- | --- |
| **sh-NKILA** | | **Sequence** | | | |
| sh-NKILA1 | | GGAGAAGTCACACGTTGATTGCTCGAGCAATCAACGTGTGACTTCTCC | | | |
| sh-NKILA2 | | GGCAGTAGGAAAGGAGAATTGCTCGAGCAATTCTCCTTTCCTACTGCC | | | |
| sh-NKILA3 | | GACTCAGTGTGGCCGAATATTCTCGAGAATATTCGGCCACACTGAGTC | | | |
| si-HIF-1α#1 | | Forward | 5′- AGAACCCAUUUUCUACUCAGTT-3′ |  |  |
|  |  | Reverse | 5′- CUGAGUAGAAAAUGGGUUCUTT-3′ |  |  |
| si-HIF-1α#2 | | Forward | 5′- GACACAGCCUGGAUAUGAATT -3′ |  |  |
|  |  | Reverse | 5′- UUCAUAUCCAGGCUGUGUCTT -3′ |  |  |
| si-HIF-1α#3 | | Forward | 5′- UUCUCCGAACGUGUCACGUTT -3′ |  |  |
|  |  | Reverse | 5′- ACGUGACACGUUCGGAGAATT -3′ |  |  |
|  | |  | | | |

| **Table S2. Primer sequences used for qRT-PCR** | | |
| --- | --- | --- |
| **Gene** | **Primers** |  |
| β-actin | Forward | 5′-AAAGACCTGTACGCCAACAC-3′ |
|  | Reverse | 5′-GTCATACTCCTGCTTGCTGAT-3′ |
| NKILA | Forward | 5′-AACCAAACCTACCCACAACG-3′ |
|  | Reverse | 5′-ACCACTAAGTCAATCCCAGGTG-3′ |
| HIF-1α | Forward | 5′- TGCAACATGGAAGGTATTGC -3′ |
|  | Reverse | 5′- TTCACAAATCAGCACCAAGC -3′ |
| GLUT1 | Forward | 5′-CATCCCATGGTTCATCGTGGCTGAACT-3′ |
|  | Reverse | 5′-GAAGTAGGTGAAGATGAAGAACAGAAC-3′ |
| HK2 | Forward | 5′-GCCATCCTGCAACACTTAGGGCTTGAG-3′ |
|  | Reverse | 5′-GTGAGGATGTAGCTTGTAGAGGGTCCC-3′ |
| PFKFB2 | Forward | 5′-AGTCCTAC-GACTTCTTTCGGC-3′ |
|  | Reverse | 5′-TCTCCTCAGTGAGATACGCCT-3′ |
| PFKFB3 | Forward | 5’-GATGCCCTTCAGGAAAGCCT-3′ |
|  | Reverse | 5’-TCCCCGACGTTGAACACTTT-3′ |
| ALDOA | Forward | 5′-AGGCCATGCTTGCACTCAGAAGT-3′ |
|  | Reverse | 5′-AGGGCCCAGGGCTTCAGCAGG-3′ |
| GAPDH | Forward | 5′-TTCCGTGTCCCCACTGCCAACGT-3′ |
|  | Reverse | 5′-CAAAGGTGGAGGAGTGGGTGTCGC-3′ |
| PGK1 | Forward | 5′-ATGTCGCTTTCTAACAAGCTGA-3′ |
|  | Reverse | 5′-GCGGAGGTTCTCCAGCA-3′ |
| PGAM1 | Forward | 5′-GGAAACGTGTACTGATTGCAGCCC-3′ |
|  | Reverse | 5′-TTCCATGGCTTTGCGCACCGTCT-3′ |
| ENO1 | Forward | 5′-GACTTGGCTGGCAACTCTG-3′ |
|  | Reverse | 5′-GGTCATCGGGAGACTTGAA-3′ |
| ENO2 | Forward | 5′-TCATGGTGAGTCATCGCTCAGGAG-3′ |
|  | Reverse | 5′-ATGTCCGGCAAAGCGAGCTTCATC-3′ |
| PKM2 | Forward | 5′-GCCCGTGAGGCAGAGGCTGC-3′ |
|  | Reverse | 5′-TGGTGAGGACGATTATGGCCC-3′ |
| PDK1 | Forward | 5′-ATCCCATCTCTATCACATGGTGTT-3’ |
|  | Reverse | 5′-GGTGCAGTTGAATACATGTAGTTG-3’ |
| LDHA | Forward | 5′-ATGGCAACTCTAAAGGATCA-3′ |
|  | Reverse | 5′-GCAACTTGCAGTTCGGGC-3′ |
| MCT1 | Forward | 5′-GTGGCTCAGCTCCGTATTGT-3, |
|  | Reverse | 5′-GAGCCGACCTAAAAGTGGTG-3′ |

**Table S3. Antibodies for Western blot**

| **Antibodies** | **Source** | **Dellution ratio** |
| --- | --- | --- |
| β-actin | Beyotime, China | 1:1000 |
| HIF-1α | Abcam, USA | 1:500 |
| VEGFA | Proteintech, China | 1:1000 |
| GLUT1 | Abcam, USA | 1:1000 |
| P53 | Abcam, USA | 1:1000 |
| HK2 | Cell Signaling Technology, USA | 1:1000 |
| PFKFB2 | Cell Signaling Technology, USA | 1:1000 |
| PFKFB3 | Cell Signaling Technology, USA | 1:1000 |
| ALDOA | Cell Signaling Technology, USA | 1:1000 |
| GAPDH | Cell Signaling Technology, USA | 1:1000 |
| PGAM1 | Cell Signaling Technology, USA | 1:1000 |
| ENO-1 | Cell Signaling Technology, USA | 1:1000 |
| ENO-2 | Cell Signaling Technology, USA | 1:1000 |
| PKM2 | Cell Signaling Technology, USA | 1:1000 |
| PDK1 | Cell Signaling Technology, USA | 1:1000 |
| PDH | Cell Signaling Technology, USA | 1:1000 |
| MCT1 | Cell Signaling Technology, USA | 1:1000 |
| ET1 | Proteintech,CHINA | 1:1000 |
| CD71 | Proteintech,CHINA | 1:1000 |
| NF-κB p65 | Cell Signaling Technology, USA | 1:1000 |
| IκBα | Cell Signaling Technology, USA | 1:1000 |
| Phospho-NF-κB p65 | Cell Signaling Technology, USA | 1:1000 |
| Phospho-IκBα | Cell Signaling Technology, USA | 1:1000 |
| secondary antibodies  （goat anti-mouse and goat anti-rabbit IgG antibody） | Beyotime, China | 1:1000 |
